# Halides with Fifteen Aliphatic C–H···Anion Interaction Sites

**DOI:** 10.1038/srep30123

**Published:** 2016-07-22

**Authors:** Genggongwo Shi, Zahra Aliakbar Tehrani, Dongwook Kim, Woo Jong Cho, Il-Seung Youn, Han Myoung Lee, Muhammad Yousuf, Nisar Ahmed, Bahareh Shirinfar, Aaron J. Teator, Dominika N. Lastovickova, Lubna Rasheed, Myoung Soo Lah, Christopher W. Bielawski, Kwang S. Kim

**Affiliations:** 1 Department of Chemistry, Ulsan National Institute of Science and Technology (UNIST), Ulsan 44919, Korea; 2 Department of Chemistry, Pohang University of Science and Technology, Pohang 790-784, Korea; 3 School of Chemistry, University of Bristol, Bristol, BS8 1TS, UK; 4 Department of Chemistry, The University of Texas at Austin, Austin, TX 78712, USA

## Abstract

Since the aliphatic C–H···anion interaction is relatively weak, anion binding using hydrophobic aliphatic C–H (C_ali_–H) groups has generally been considered not possible without the presence of additional binding sites that contain stronger interactions to the anion. Herein, we report X-ray structures of organic crystals that feature a chloride anion bound exclusively by hydrophobic C_ali_–H groups. An X-ray structure of imidazolium-based scaffolds using C_ali_–H···A^−^ interactions (A^−^ = anion) shows that a halide anion is directly interacting with fifteen C_ali_–H groups (involving eleven hydrogen bonds, two bidentate hydrogen-bond-type binding interactions and two weakly hydrogen-bonding-like binding interactions). Additional supporting interactions and/or other binding sites are not observed. We note that such types of complexes may not be rare since such high numbers of binding sites for an anion are also found in analogous tetraalkylammonium complexes. The C_ali_–H···A^−^ interactions are driven by the formation of a near-spherical dipole layer shell structure around the anion. The alternating layers of electrostatic charge around the anion arise because the repulsions between weakly positively charged H atoms are reduced by the presence of the weakly negatively charged C atoms connected to H atoms.

The structure of an anion that is surrounded exclusively by multiple C_ali_–H groups (often considered to be hydrophobic) is hardly anticipated without the presence of any other binding sites such as cations or polar residues. Because the C_ali_–H···A^−^ interaction is weak, anion binding by the C_ali_–H groups is generally enhanced through the incorporation of additional binding sites to the anion. Indeed, though C–H···A^−^ type interactions^1^ are ubiquitous in nature, C_ali_–H groups are rarely used as H-bond donors in synthetic receptors. Nevertheless, such interactions are essential to the overall stability of complexes of proteins and DNA as well as various organic transformations and the transition states of diverse catalytic cycles^2^,^3^. The design of anion receptors with C_ali_–H donors constitutes a useful opportunity to tailor molecular recognition phenomena^4^,^5^. It is, however, often difficult to directly probe C_ali_–H···A^−^ interactions due to relatively low binding strengths, although there are some reports about interactions between anions and multi C_ali_–H donors^6^,^7^,^8^. In some applications (e.g., anion templated assembly)^9^,^10^,^11^, aryl C–H groups are used to enhance C–H···A^−^ interactions. In comparison, the C–H bonds found in nonpolar alkanes are relatively less acidic, though the corresponding interactions with anions may be increased through the incorporation of electron-withdrawing groups.

The first charge-neutral systems of highly fluorinated receptors with aliphatic methylene groups were polarized by neighboring electronegative O and F atoms^12^. Computational investigations also played a vital role in understanding the strength of C_ali_–H···A^−^ H-bonds^13^. Gas-phase C_ali_–H···A^−^ hydrogen bonding was reported for adducts of anions and a resorcinarene cavitand, which was shown to adopt a bowl-shaped cavity that provided up to four convergent C–H groups activated by adjacent oxygen atoms^6^. Other examples of host-guest complexes in solution stabilized by C_ali_–H hydrogen bonding appear to be limited to a palladium complex^7^. Moreover, 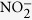 was found to stabilize the ring conformations adopted by sugars via H-bonding to three axially positioned C_ali_–H groups^14^. It has also been shown that the aliphatic bridge methanetriyl and methylene protons in imidazolium-based receptors are polarized by the positive charges and thus are more acidic when compared to their neutral analogues^15^,^16^,^17^. In this regard, it is possible to design a structure having a large number of C–H···A^−^ interaction sites towards an anion.

Herein, we disclose the synthesis and study of a bis-imidazolium host bearing acidic C_ali_–H groups. The methyl and methylene moieties in this host form C_ali_–H···A^−^ interactions with various anions. In particular, we report that bisimidazolium and tetraalkylammonium based hosts drive the formation of multideca to pentadeca binding sites with halide anions exclusively through C_ali_–H···A^−^ interactions.

## Results

**Figure 1:**
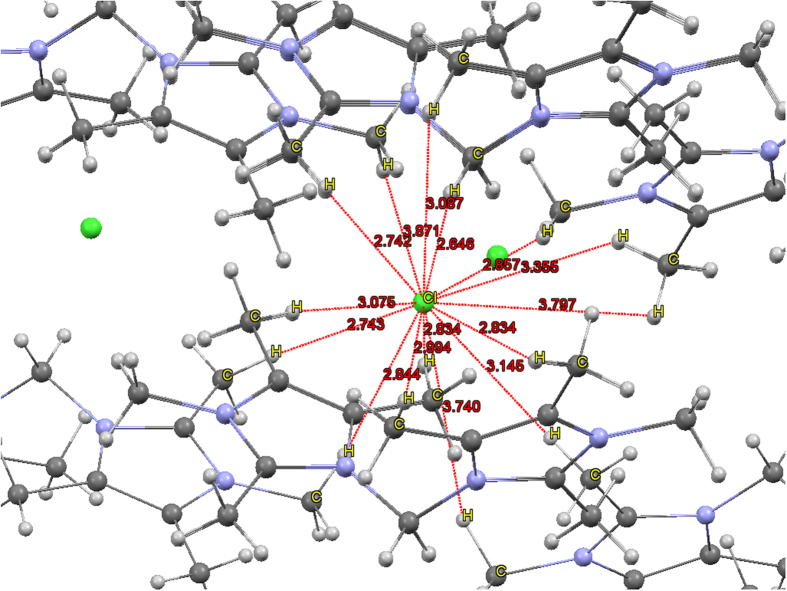
C–H···Cl^−^ interactions of [1](Cl)_2_ showing 15 binding sites for Cl^−^ (drawn using Mercury^18^). The direct Cl···C binding distances (Å) are denoted in red color (green: Cl^−^, blue: N, dark gray: C, light gray: H).

1,1′-methylenebis(2,2′,3,3′,4,4′ ,5,5′ -octamethylimidazolium) di-hexafluorophosphate [**1**](PF_6_)_2_ was synthesized and characterized using NMR spectroscopy (Figures S1-1 to S1-3, See Supplementary Information (SI), Section 1). Single crystals of [**1**](Cl_2_) were grown under anhydrous conditions. Single crystal X-ray diffraction analysis (Figure S2-1) showed the structure featuring C_ali_–H···Cl^−^ interactions between the bridging methylene H atoms of multiple bisimidazolium guests, whose hydrogens were found in the differential Fourier map and refined with isotropic displacement coefficients U(H) = 1.2U. The Cl^−^ anion appeared to be surrounded by multiple C_ali_–H groups (Fig. 1; drawn using the Mercury software package^18^). Due to the lack of local symmetry, the number of direct binding sites (in the primary binding shell) is described in a broad sense by adopting a criterion to determine the number of H atoms which are favorably interacting directly with Cl^−^. This can be inspected using Voronoi based nearest neighbor search^19^. Namely, a certain H atom of H_x_ belongs to the primary binding shell only when there is no other atom within the spherical surface centered at the midpoint of H_x_ and Cl with a diameter of the H_x_···Cl distance. This condition eliminates some of the H atoms that might otherwise be erroneously assigned to the primary binding shell.

According to IUPAC, “the hydrogen bond is an attractive interaction between a hydrogen atom from a molecule or a molecular fragment X–H in which X is more electronegative than H, and an atom or a group of atoms in the same or a different molecule, in which there is evidence of bond formation”. Evidence for bond formation requires fulfillment of several criteria^20^,^21^. The bond length is likely to be less than or at least comparable to the sum of van der Waals (vdW) radii of the two bonding atoms where the anisotropic property^22^ of the vdW radius and the variance of vdW radius are taken into account due to the contacting environment^23^. In this work, we use the following parameters: vdW radius^23^ of H/C/Cl^−^: 1.20/1.7–1.77/1.81 Å, the sum of vdW radii of H and Cl^−^ r^vdW^_Cl···H_: 3.01 Å and the sum of vdW radii of CH and Cl^−^ r^vdW^_Cl···(H)C_: 4.12 Å in consideration of C–H distance ranging 1.105–1.11 Å. A typical hydrogen bond is generally considered as a weak electrostatic chemical bond between a negatively charged atom and a positively hydrogen atom bound to another negatively charged atom. It also shows some characteristics of non-covalent interaction that does neither form nor break actual bonds, similar to dipole-dipole interactions or charge-dipole interactions.

Then, in our crystal structures, we find that these H_x_’s are considered to bind Cl favorably through either H-bonds {whose distances are shorter than or comparable to *r*^vdW^_Cl···H_: 3.01 Å or *r*^vdW^_Cl···(H)C_: 4.12 Å} or H-bonding-like interactions, with favorable H-bonding/binding angles Cl···H_x_-C_ali_ (*θ* ≥ 100°) (Table 1). It should be noted that binding needs not to be bonding. In contrast to H-bonding, the H-bonding-like interaction does not mean a formation of such a short H-bonding, but includes H-bonding-like cases with the A^−^-H binding distance beyond the proper H-bonding distance as well as a favorable H-bonding/binding angle showing significant attraction (not repulsion). As such, the Cl···H_x_-C_ali_ binding energies (BEs) in [**1**](Cl)_2_ are not small (1–3 kcal/mol for the Cl^−^···H-CH_3_ cases; 64–83 kcal/mol for the Cl^−^···H-CH_2_NH_3_^+^ cases).

From the analysis of binding sites for Cl^−^ in bis-tetramethylimidazolium complexes, we were pleasantly surprised to find that one of two kinds of Cl^−^ has 15 binding sites via H-bonds and H-bonding-like interactions, ten strong H-donors (#1-10, shorter than *r*^vdW^_Cl···H_ (=2.9–2.97 Å)), 1 moderate H-donor (#13), and four weak H-donors (#11, 12, 14, 15) (Fig. 1, Table 1a). In terms of the Cl^−^···C distances (*d*_Cl···C_), twelve C atoms were within H-bond distance from Cl^−^ (4.01–4.08 Å), in consideration that *r*^vdW^_Cl···(H)C_: 4.01–4.08 Å (note that one C atom was interacting in a bidentate manner with the Cl^−^ via its two H atoms #11,12), while additional two C atoms (#14,15) were positioned at relatively long distances (4.71, 4.83 Å). The C_ali_–H···Cl^−^ angles (*θ*) were measured to be in all circumstances greater than 148° except for the bidentate case (129/124° and 100/100° in X-ray/DFT) by the two H atoms (#11, 12) attached on the same C atom at *d*_Cl···C_ = 4.05 Å. Namely, eleven C atoms involved in strong bidentate H-bonds were in agreement with the formation of bona fide H-bonds. In addition, one C atom involved in bidentate C_ali_–H···Cl^−^ interactions with its two H atoms was considered to have weak H-bonding-like interactions (*θ*: 129° and 100°) because the *d*_Cl···H_ (= 3.35, 3.80 Å) distances are somewhat long even though *d*_Cl···C_ (= 4.05 Å) ≈ r^vdW^_Cl···(H)_ (= 4.01–4.08 Å). Nevertheless, these bidentate H-bonding-like interactions could be considered as bidentate H-bonds or at least bidentate H-bonding-like bindings. Further, two weak uni-dentate H bonding-like interactions at relatively long distances (*d*_Cl···C_ = 4.71/4.83 Å) but with favorable angles (*θ*: 153°/177°) can also be taken into account as direct binding sites. Then, Cl shows fifteen binding sites (thirteen uni-dentate and one bidentate H-bonding or H-bonding-like interaction sites) by the C_ali_–H groups in seven molecules of [**1**] surrounding the Cl^−^ anion. Furthermore, each C_ali_–H···Cl^−^ binding for the given d_Cl···H_ and *θ* shows adequate BEs (1–3 kcal/mol in Table 1), which were calculated at the B97D/aug-cc-pVDZ level of theory for the binding between Cl^−^ and a group with the formal formula of CH_4_. Here, the methane-like group features the geometry adopted by each C_ali_–H group as well as the typical –CH_3_ geometries for the remaining three H atoms. Since the position of the H atoms as determined by using X-ray crystallography is not sufficiently accurate enough for computing BEs, the DFT optimized CH distances were used to obtain the DFT BEs. We also considered the Cl^−^···H_3_C-NH_3_^+^ interaction, which gives strong BEs (64–83 kcal/mol) for all the fifteen cases due to the strong electrostatic interactions between negatively charged Cl^−^ and positively charged H_3_C-NH_3_^+^. In all the above cases of fifteen H atoms, no other atom exists within the sphere having the diameter from each H atom to Cl^−^, and thus, the fifteen H atoms directly bind Cl^−^ as Voronoi nearest neighbors.

Although we classified the above binding interaction as the H-bonding and H-bonding-like interactions, even the latter show most features of H-bond addressed in the IUPAC provisional recommendation (criteria and characteristics)^20^,^21^ except for the H-bond distance criterion. Although the highest occupied molecular orbital (HOMO) barely shows partial bonding orbital character (since only weak bonding-type orbital mixing exists: see Figure S3-1), the Cl^−^···H-C_ali_ is mainly an electrostatic interaction between positive and negative charges where the positive charge of H is enhanced due to the polarization through the H-C_ali_ bond. In the model system of Cl^−^···H-CH_3_, the induction and electrostatic energies based on DFT (PBE0AC/aug-cc-pVDZ xc functional; basis set with ALDA xc kernel) are dominant for the binding in the fully optimized structure (∆*E*_total_ = −3.51; ∆*E*_elst_ = −2.40; ∆*E*_ind_ = −2.37 kcal/mol; ∆*E*_disp_ = −1.68; ∆*E*_exch_ = 3.81 kcal/mol at *d*_Cl···H_ = 2.705 Å and *θ* = 180°). Even with a highly increased distance between H and Cl, the induction energy gives significant contribution for binding: ∆*E*_total_ = −2.33; ∆*E*_elst_ = −0.36; ∆*E*_ind_ = −0.95; ∆*E*_exch_ = −0.36 kcal/mol for *d*_Cl···H_ = 3.871 Å (more information in Table S3-2). It also strongly depends on the angle. Furthermore, the electrostatic interaction between Cl^−^ and CH_3_-NH_3_^+^ clearly shows a very strong ∆*E*_elec_. Also, due to the N atom in the imidazolium moiety to which H-C_ali_ is bonded, H-C_ali_ is polarized enough (more positive charge on the H atom in imidazolium-CH_3_ (+0.247) than in the methane model (+0.218). All of the Cl^−^···H-C_ali_ angles deviate slightly from 180° but, by considering flexibility of the range of H-bond angles, the angles in the 15 H atoms cases are in favor of significant interaction energies. In addition, if the angle reduces from 180° to 100°, the H_x_-C (H_x_: hydrogen interacting with Cl^−^) bond distance decreases from 1.108 to 1.102 Å, while Cl^−^···H_x_ distance increases from 2.705 to 3.5 Å. Namely, as the angle is close to 180°, the Cl^−^···H_x_ interaction energy becomes stronger, showing the significant angle-dependence of the H-bonding characteristics.

**Figure 2:**
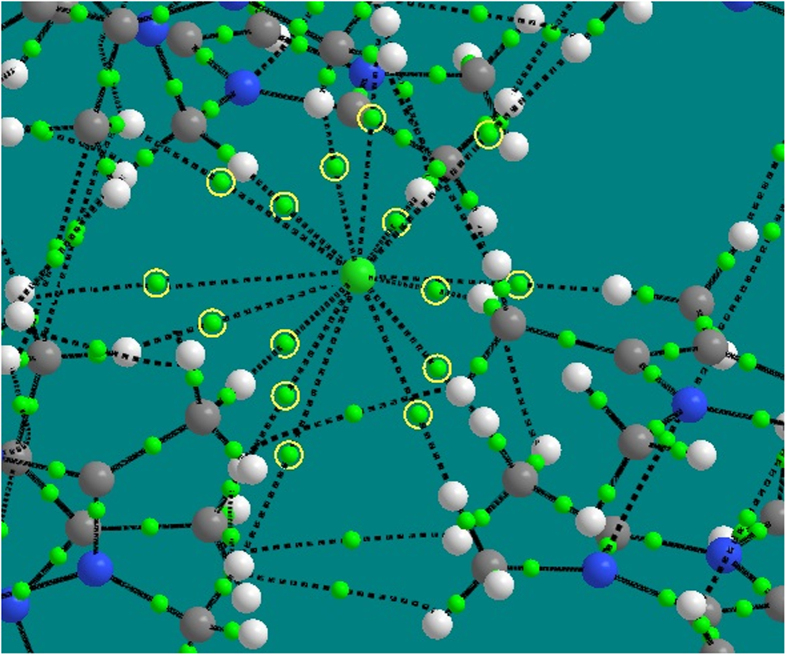
Fifteen bond critical points (green spheres highlighted with yellow circle) for Cl^−^···H H-bonding-like interactions in the crystal structure of [**1**](Cl)_2_ along with bond paths (dashed lines). (green: Cl^−^, blue: N, dark gray: C, light gray: H).

H-bonds may be studied via electron density topology by using the bond path that connects the H and Y atoms through a (3, –1) critical point^24^. The Cl^−^···H–C_ali_ interactions in the CH_4_–Cl^−^ and NH_3_CH_3_^+^···Cl^−^ models as well as the crystal structure of [**1**](Cl)_2_ have H-bond-like characteristics based on both the IUPAC recommendation and the Koch-Popelier definition (ρ(r) = 0.002–0.040 au and 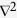
ρ(r) = 0.024–0.139 au)^25^ for conventional hydrogen bond (Fig. 2; more information of quantum theory of atoms in molecules (QTAIM) analysis and plot of noncovalent interaction (NCI) regions are given in SI: Sections 3.3 and 3–4, Tables S3–4 and S3-5, and Figure S3-2). These interactions are electrostatic in nature (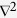
ρ (r) > 0), as described earlier. Moreover, non-covalent interaction analysis in the [**1**](Cl)_2_ crystal structure highlights that Cl^−^ is stabilized by strong H-bonds as well as strong ionic electrostatic interactions (d_Cl···Hx_ ≤ 3.15 Å in X-ray structure) and weak H-bonding-like interactions (Figure S3-2). Overall, the system here fits some criteria of H-bonding.

In contrast to the highest coordination of metal cations^26^,^27^, the high number of binding sites for anions has not been explored in detail to the best of our knowledge. Here, we particularly focus our attention on halide anion recognition because large-sized molecular anions are somewhat complex in host-guest interactions. This anion recognition by hydrophobic C_ali_-H groups is conceptually similar to the cation recognition of hydrophobic aromatic rings, but the origin of noncovalent interactions are quite different (H-bond-like interaction vs. cation-π interaction^28^). In general, halide anion coordination favors the formation of asymmetric structures such as those that are tweezer-like^29^,^30^,^31^, tripodal^32^, or cone-like^33^,^34^,^35^,^36^ because the excess electron density needs to be present in a large vacant space around the halogen to improve stabilization. Moreover, the larger the vacant space, the more stable the excess electron density due to quantum confinement effects. Upon binding by few or several ligands through the usual anion···H bonding interaction, the strong charged or ionic H-bonds formed between an anion and the ligands tend to squeeze the vacant space around the anion into a certain solid angle of the non-bonded empty space. This is the reason why a halide anion hydrated by a few number of water molecules has an asymmetric structure for which the water is non-spherically clustered on one side of the surface of the halide anion, i.e., a vacant space around the non-bonded surface of the anion (i.e., within a certain solid angle)^37^. This in turn is the reason why the tweezer-, tripodal- or cone-like structures adopted by many host-guest supramolecular systems are widely exploited for the design of anion receptors^38^. Such non-symmetric structures are natural as long as the coordination number of the anion···H bonds is not large. Thus, so far, the use of anion···H interactions have rarely resulted in complexes that exhibit spherical coordination structures. However, when the coordination number increases to a high number, the vacant space required to stabilize the excess electron is no longer available. Once the excess electron density is squeezed, the tiny vacant space on a small solid angle would make the excess electron unstable due to the quantum confinement effects. Then, the optimal option is to let the excess electron density stay uniformly distributed over all the near-spherical surface of the anion. In such a case, the anion-ligand interaction needs to be weak so that an empty spherical shell can be present between the anion and ligands. The positive charges of the ligand atoms should be small so as not to be too repulsive between the neighboring ligands surrounding the anion. Thus, weakly positively charged H atoms in C_ali_–H groups would be a good choice. In this regard, herein we describe the use of weak C_ali_–H···A^−^ interactions where C should not be strongly negatively charged.

**Figure 3:**
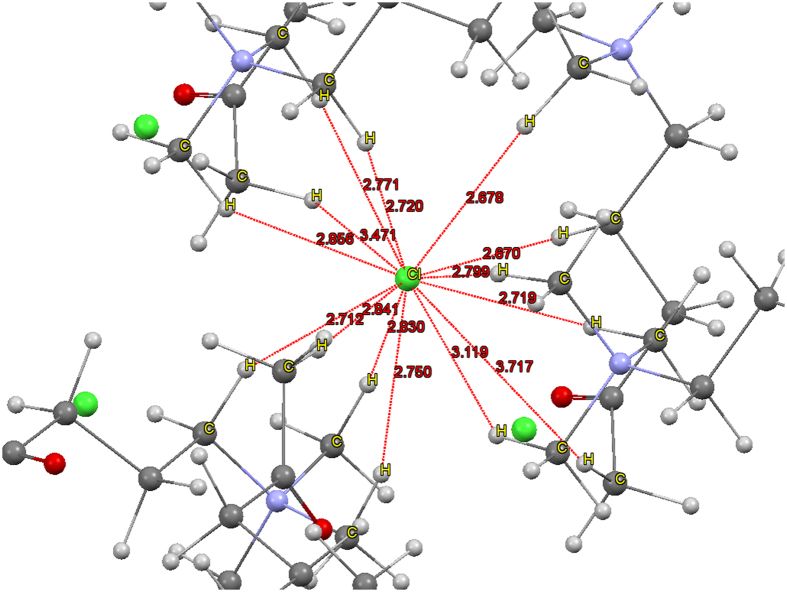
Fourteen C–H···Cl^−^ interaction sites in crystal structures of BUXTOD systems^39^, where only H positions were optimized at the B97D/cc-pVDZ level. The direct Cl···C binding distances (Å) are denoted in red color (green: Cl^−^, blue: N, red: O, dark gray: C, light gray: H).

One could expect that other complexes with such a high number of binding sites could also be possible. We searched the Cambridge Crystal Structure Database (CCSD) for such high number of binding sites similar to those described herein. Therein, we indeed found 14 binding sites for anions in the tetraalkylammouniun complexes (BUXTOD)^39^ by C_ali_–H···anion interactions (with twelve strong to moderate H-bonds and two relatively weak H-bonding-like interactions) (Table 1b, Fig. 3, and SI), where the Cl^−^ anion was surrounded by six tetraalkylammoium complexes.

**Figure 4:**
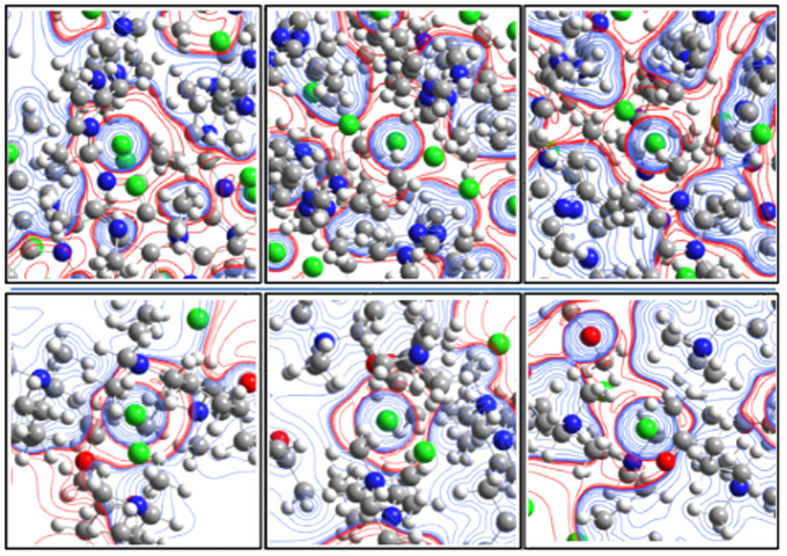
Concentro-spherical shells composed of alternating +/− electrostatic potential regions (light-red/light-blue contours) for the C_ali_–H···Cl^−^ interactions of [**1**](Cl)_2_ (upper three panels) and the BUXTOD complex involving tetraalkyl-ammonium cations (lower three panels) at the B97D/cc-pVDZ level (Each three panels denote the +/− electrostatic potential contour maps on the three perpendicular planes with respect to the Cl^−^ anion. Isodensity surface value: 0.01 au; Isovalue for contour; 0.008 au. (Cl: green, O: red, N: blue, C: dark grey, H: light grey).

Upon close inspection of all the above H-bonding and H-bonding-like interaction driven multi-coordination structures, the respective anions were surrounded by weakly positively charged H atoms contained within CH_3_ groups that were bonded to the N atoms, where the C atom was slightly positively charged due to the ammonium group. Thus, weakly positively charged H atoms surround the anion nearly spherically, and then the weakly negatively charged C atoms surround the region of positively charged H atoms. The nearly concentric structures showing alternating +/− electrostatic potential shells are formed (Fig. 4).

Such nearly centrospherical shell structures containing multiple C–H···A^−^ interactions may be contrasted with highly asymmetric structures^40^ of most halide anion receptors. Since H-C-N- residues show small charges due to cancellation between positive and negative charges of the C and N atoms, the repulsions between the positive charges are reduced by the presence of anionic charges in the next spherical shell layer. In this way, such C_ali_–H···A^−^ interactions may be considered to be prevalent, despite that it has never been disclosed previously. Indeed, we note that the structures coordinated by more than eleven C_ali_–H groups can be found in CCSD (ACHOLC, etc)^39^. Additionally, one may find dodeca-coordination to halide anions from GUVLEP, IWEMED, IWEMAZ^13^,^41^, where only one halide anion is inside a large template molecule, but not surrounded by a number of solvent molecules [*d*_Cl···H_ = 2.88–3.13 Å, *d*_Cl···C_ = 3.65–3.75 Å, *θ*_Cl···H–C_ = 121–132°; *d*_I···H_ = 3.35–3.30 Å, *d*_I···C_ = 3.88 Å, *θ*_I···H–C_ = 117–120°]. In consideration of small angles, this dodeca coordination somewhat reflects a caged structure, i.e., strained coordination, instead of H-bonding. However, it may still be labeled as a dodeca-coordination complex, with centrospherical shells that feature C_ali_–H···A^−^ interactions and contain alternating regions of +/− electrostatic potential.

## Discussion

Our study finds that the Cl^−^ anions in the bisimidazolium complex have pentadeca binding sites exclusively by C_ali_–H groups. The electrostatic potential maps feature nearly concentro-spherical shells with alternating +/− electrostatic interactions, quite different in structure from many well-known H-bond complexes for anions. These intriguing C_ali_–H···A^−^ interactions have not been properly recognized previously. The positively charged hosts render the aliphatic C–H moieties relatively acidic and thus increase their binding affinities for anions. Collectively, the results described herein may give rise to new classes of aliphatic hosts that display selectivity toward anions via tight control of cavity geometry and acidity of their respective H-bond donors. We also note that complexes of tetradeca binding sites and other multiple binding sites show nearly concentro-spherical shells depicting alternating +/− electrostatic potential for the C_ali_–H···A^−^ interactions.

Here, we discuss the number of binding sites as compared with the coordination number (CN) which are well defined in inorganic chemistry. In solid and liquid states materials, the CN is also often used, differently from the terminology used in inorganic chemistry. Indeed, the CN has been a widely used terminology in various branches of science. The definition of CN originates from mathematics, where it means the number of equivalent hyperspheres in n dimensions that can touch an equivalent hypersphere without any intersections^42^. It is also called the contact number, ligancy, kissing number, or Newton number. Newton was the first to define the CN. The CN in 3 dimensions is 12 in hexagonal close packing. As molecules and metals of different sizes tend to pack in compact structure based on intermolecular interactions, Werner proposed the structures for coordination compounds containing complex ions whereby a central transition metal atom is surrounded by neutral or anionic ligands^43^. In inorganic coordination chemistry, the CN refers to the number of σ-bonds formed between the ligands and the central atom. However, in terms of IUPAC terminology, the CN of a specified atom in a chemical species is the number of other atoms directly linked to that specified atom^44^. For molecules and polyatomic ions, the CN of an atom is the number of the other atoms to which it is bonded. However, these kind of bonds cannot often be clearly defined in solid state crystals and the neighboring atoms may not be at the same distance; thus, in material science, the CN is the number of direct neighbors to a given atom. In quasicrystals, liquids and other disordered systems, a more general expression is required. The first and second coordination numbers are defined as the number of neighbors of a central atom in a molecule/ion using the first and second minima of a radial distribution function, respectively. In liquid, due to time dependent structural changes, the statistically averaged coordination shells are generally used and the average CN is often a fractional number (e.g., first CN of liquid water is 4.7)^45^,^46^. Thus, in a broad sense, the CN can be defined as the number of atoms, ions, or binding sites directly surrounding a central atom or ion in a host-guest complex, a condensed form of matter, or a liquid.

Though the definition might not be clear in certain cases, it is assumed that each binding event needs to show significant positive (i.e., attractive) BE associated with noncovalent interaction, noncovalent bonding, or electrostatic interaction. The van der Waals (vdW) interactions are generally not considered in counting CNs. Coordination phenomena often include cation-anion interactions, hydrogen bonding, and π interactions^47^,^48^,^49^. In addition, indirect or secondary coordination should be excluded from direct or primary coordination in counting CNs.

When the local coordination does not have high symmetry, the direct coordination can be considered as a case where a given coordinating atom (or site) does not have any other atoms in the sphere centered on the midpoint between the given site and the coordinated atom. This can be analyzed by measuring the angle *θ* between two vectors constructed from any other atom to the coordinating site and the coordinated site. If any other atom has the angle *θ* > 90°, the assumed coordinating atom/site should be removed from the direct coordination. If all angles *θ* obtained for all other atoms are less than 90°, it means that all of them are out of the sphere, and so this case is considered to be directly coordinated. In this way, the obtained CN is equivalent to the Voronoi-based CN nearest neighbors^50^ in 3 dimensions. If any site is within such a sphere, the coordinating site cannot be the direct or primary site because that site is more primary than the coordinating site. In this way, the direct/primary and indirect/non-primary sites can be properly distinguished even in the cases of amorphous or liquid structures as well as the crystal structures that are not sufficiently symmetric to define the first and second coordination shells.

Although examples of complexes that feature multiple coordination numbers have been widely observed, cases of very high coordination are rare. Structures with very high coordination numbers have been found with actinide-based cations, such as [U(BH_4_)_4_]^51^. A recent theoretical prediction of pentadeca-coordination for PbHe_15_^2+^, which has a large metal cation and many small ligands^26^, could have catalyzed the search for pentadeca-coordination complexes. Indeed, a pentadeca-coordinated complex was experimentally observed from thorium aminodiboranate [Th(H_3_BNMe_2_BH_3_)_4_]^27^. The crystal features seven (bidentate) double H-bridge that contain Th···H–B H-bonds with an almost perpendicular bond angle (~100°) and one single H-bridge containing a uni-dentate Th···H–B H-bond with a bond angle of ~103°. These bond angles are far from the optimal linear H-bonding (180°), and so each H-bond is different from those typically found in organic compounds. The pentadeca-coordination structure originates from the tetradeca-coordinated bicapped hexagonal antiprism of U(BH_4_)_4_, its derivatives [U(BH_4_)_4_·OMe_2_, U(BH_4_)_4_·OEt_2_, U(BH_4_)_4_·2OC_4_H_8_]^52^,^53^, and analogues which utilized large metal complexes (M: Th, Pa, etc.)^54^,^55^ and BH_4_ groups.

The U(BH_4_)_4_ crystal showed a distorted-octahedral arrangement about the U(IV) center which was coordinated by twelve H atoms comprised of six bridging borohydride groups (two H atoms each) and two additional H atoms of two terminal borohydride groups. The bridging groups have double H-bridges (bidentate H-bonding) with bond angles (∠U···H–B) of 96–99°, while the terminal groups feature triple H-bridges (tridentate H-bonding) with bond angles of 82–86° (which are much smaller than the typical H-bond angle recommended by IUPAC (18, 19)). All the above pentadeca and tetradeca-coordination complexes exploited tridentate and bidentate binding modes of B–H···M interactions which showed nearly perpendicular H-bond angles; again, these are not typical H-bonds, but electrostatically-driven interactions that were assisted by multi-dentate metal H-bonds. Nevertheless, such cases were considered as H-bonding (instead of H-bonding-like interactions).

In comparison, dodeca-coordination for cations of cerium, uranium and thorium with bi-dentate nitrate ion ligands, Ce(NO_3_)_6_^2−^, U(NO_3_)_6_^2−^ and Th(NO_3_)_6_^2−^ 56, where the metal is bound by two O atoms of each nitrate ligand, are relatively common. Such interactions are possible because the cuboctahedron (O_h_) geometry (either in a local environment or in crystal symmetry) can structurally allow dodeca-coordination as the cation favors spherical coordination towards many ligands^57^. However, in all these cases, the angles of metal···O–N bonds is bent (96–98°, far from linear) and the positively charged N atoms reduce the repulsions between O atoms in adjacent (NO_3_)^2−^ groups. The bent angle of the metal···O–N bond could not be considered to be a bona fide cation–O covalent bonding type interaction; rather, it may be considered as an electrostatically favored interaction that is driven by packing. It should be noted that the structures of UCp_4_ and ThCp_4_ are generally considered to have the coordination number of 12^58^,^59^,^60^.

Overall, CN can be explicitly used in inorganic chemistry community. However, since Newton initially used CN as the number of nearest neighboring sites, the CN has been indeed extended to counting the number of contacting sites in solvent structure in physical chemistry and statistical mechanics. We may adopt a conservative criterion to determine the number of H atoms in direct contact with Cl^−^. However, the binding sites is rather restricted to the favorable interaction such as H-bonding-like interactions with favorable bond angle. In water, it is generally accepted^46^ that the CN is 4.7, while the number of binding sites is only 4.0. In this regard, the CN in a broader sense (though it is hardly to be acceptable in inorganic chemistry) could be considered as 15 in our crystal structure. The number of binding sites is also 15 because all the direct contacting sites show significant binding energies with favorable C-H orientations.

## Methods

### General considerations

**Figure 5:**
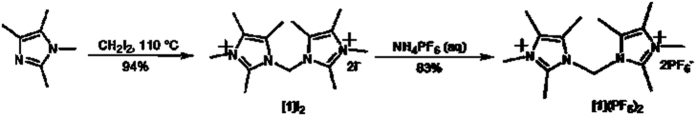
Synthesis of [1](PF_6_)_2_.

All reactants and solvents were purchased from commercial sources and used without further purification. All products were characterized using ^1^H and/or ^13^C NMR spectroscopy, as performed on a Bruker Advance DPX500 (500 MHz) spectrometer at 298 K. All new compounds were also characterized by mass spectroscopy. Low resolution mass spectra were obtained on Bruker 1200 Series & HCT Basic System. High resolution mass spectra were obtained on a Waters Xero G2-XS Q-ToF mass spectrometer (Fig. 5).

### Synthesis of 1,1′-Methylenebis[(2,2′,3,3′,4,4′,5,5′-octamethylimidazolium)] Diiodide [1](I)_2_

1,2,4,5-Tetramethylimidazole (1.24 g, 10 mmol) and diiodomethane (1.35 g, 5 mmol) were heated at 110 °C in a sealed tube overnight. The resulting mixture was filtered and washed with dichloromethane several times to afford [**1**](I)_2_ as a brown solid. Yield: 2.44 g, 94%. ^1^H NMR (*d*_*6*_-DMSO, 500 MHz), δ 6.57 (s, 2H, C*H*_2_), 3.69 (s, 6H, NC*H*_3_), 2.70 (s, 6H, N = CC*H*_3_N), 2.23 (s, 6H, NCC*H*_3_ = C), 2.08 (s, 6H, NCC*H*_3_ = C). ^13^C NMR (*d*_*6*_-DMSO, 125.7 MHz), δ 144.66, 127.24, 124.87, 54.97, 32.68, 11.02, 8.63, 8.31. LRMS (ESI^+^, MeCN), *m/z* calcd. for [2M – I]^+^ 905.500, found 905.070; *m/z* calcd. for [M – I]^+^ 389.298, found 388.914.

### Synthesis of 1,1′-Methylenebis[(2,2′,3,3′,4,4′,5,5′-octamethylimidazolium)] Dihexafluoro-phosphate [1](PF_6_)_2_

To an aqueous solution of [**1**](I)_2_ (2.44 g, 4.7 mmol) was added a saturated aqueous solution of NH_4_PF_6_ (3.06 g, 18.8 mmol) at room temperature. A precipitate formed which was subsequently collected by filtration and washed with water to afford [**1**](PF_6_)_2_ as a light brown solid. Yield: 2.16 g, 83%. ^1^H NMR (CD_3_CN, 500 MHz), δ 6.15 (s, 2H, C*H*_2_), 3.61 (s, 6H, NC*H*_3_), 2.53 (s, 6H, N = CC*H*_3_N), 2.20 (s, 6H, H_3_CNCC*H*_3_ = C), 2.04 (s, 6H, -CH_2_NCC*H*_3_ = C). ^13^CNMR (CD_3_CN, 125.7 MHz, **I**), δ 145.83 (N = CCH_3_N), 129.44 (H_3_CN-CCH_3_ = C), 126.87 (-CH_2_N-CCH_3_ = C), 56.41 (CH_2_), 33.77 (NCH_3_), 11.88 (N = CCH_3_N), 9.50 (-CH_2_N-CCH_3_ = C), 9.12 (H_3_CN-CCH_3_ = C). HRMS (ESI^+^, MeCN), *m/z* calcd. for [2M – PF_6_]^+^ 959.3240, found 959.3247; *m/z* calcd. for [M – PF_6_]^+^ 407.1799, found 407.1828; *m/z* calcd. for [M – 2PF_6_]^2+^ 131.1078, found 131.1071.

## Additional Information

**How to cite this article**: Shi, G. *et al*. Halides with Fifteen Aliphatic C-H···Anion Interaction Sites. *Sci. Rep.* **6**, 30123; doi: 10.1038/srep30123 (2016).

## Supplementary Material

Supplementary Information

## Figures and Tables

**Table 1:** Cl···H and Cl···C distances (*d*_Cl···H_, *d*_Cl···C_ in Å), H-binding angles (*θ*: ∠Cl···H–C in degree), and binding energies (BE in kcal/mol) for complexes of (**a**) bis-imidazolium and (**b**) tetraalkylammoinium^a^.

a					b			
#	*d*_Cl···C_	*d*_Cl···H_	*θ*	BE	*d*_Cl···C_	*d*_Cl···H_	*θ*	BE
1	3.574(3)	2.65(3)/2.51	167(2)/164	2.0	3.730	2.81/2.71	160/153	2.5
2	3.628(4)	2.74(3)/2.53	175(3)/173	2.5	3.756	2.87/2.75	151/152	2.3
3	3.648(4)	2.86(4)/2.67	156(3)/148	1.8	3.757	2.81/2.67	168/168	2.9
4	3.695(4)	2.74(4)/2.60	173(3)/166	2.5	3.762	2.96/2.72	150/158	2.7
5	3.761(3)	2.83(3)/2.69	163(3)/164	2.5	3.764	2.73/2.68	170/170	2.9
6	3.763(4)	2.84(4)/2.66	171(3)/176	2.8	3.767	−/2.72	−/159	2.7
7	3.780(3)	2.83(3)/2.70	165(3)/166	2.6	3.791	2.61/2.77	162/153	2.6
8	3.940(4)	3.09(4)/2.96	148(3)/148	2.0	3.822	2.87/2.80	150/154	2.6
9	3.944(4)	2.99(3)/2.85	172(3)/171	2.7	3.822	2.94/2.83	159/150	2.5
10	4.028(4)	3.08(3)/2.93	174(2)/173	2.6	3.858	3.05/2.86	143/151	2.5
11	4.046(4)	3.35(4)/3.33	129(2)/124	1.1	3.940	3.03/2.84	168/175	2.8
12	4.046(4)	3.80(4)/3.72	100(2)/100	1.0	4.069	3.05/3.12	149/145	2.0
13	4.114(4)	3.15(3)/3.00	179(2)/178	2.6	4.280	3.36/3.47	156/131	1.5
14	4.709(4)	3.74(3)/3.67	153(2)/157	1.4	4.553	3.46/3.72	154/134	1.3
15	4.828(4)	3.87(3)/3.73	177(3)/175	1.6				

^a^All the atom positions excluding H were fixed to the corresponding positions that were determined by X-ray crystallography. As for the H atom positions, both the X-ray and the B97D/cc-pVDZ optimized geometries are reported as X-ray(left)/B97D(right) in the columns of *d*_Cl···H_ and *θ*, because in the original X-ray data one of H positions was missing and some of the X-ray H positions were not reliable enough due to too long/short CH bond lengths and unreliable bond angles due to the uncertainty in resolution. Standard uncertainties (s.u.) of all contact distances and angles for (a) are in parentheses, while those for (b) are not available (ref. 38). The natural bond orbital charges of atoms (q in au) are: (a) q_Cl_ = −0.904; q_H_ = 0.18–0.26; q_C_ = ~0.2(1), −0.3(6, 7), −0.6(2–5); (b) q_Cl_ = −0.859; q_H_ = 0.21–0.26; q_C_ = ~−0.4 (−0.2, −0.7). The BEs were estimated for the Cl^−^···CH_4_ interaction, where the C of CH_4_ had the X-ray geometry of C_ali_ for each C_ali_–H group and all the H atoms were at the B97D optimized geometry with the typical –CH_3_ structure for the remaining three H atoms. The optimally computed bond distance *d*_Cl···H_ for the pure Cl^−^···CH_4_ interaction is 2.726 Å and the BE is 3.04 kcal/mol. The vdW distance for Cl···H-C and Cl···H interactions are *r*^vdW^_Cl···H-C_ = 4.01–4.08 Å and *r*^vdW^_Cl···H_ = 2.9–2.97 Å, respectively.
