# Supplementary material for: Halides with Fifteen Aliphatic C–H···Anion Interaction Sites
Source: Sci Rep. 2016 Jul 22;6:30123. doi: 10.1038/srep30123 (PMC4957075; doi:10.1038/srep30123)
Supplement: Supplementary Information [file srep30123-s1.pdf]

Supplementary Information

Halides with Fifteen Aliphatic C–H···Anion Interaction Sites

Genggongwo Shi[1,2]*, Zahra Aliakbar Tehrani[1]*, Dongwook Kim[1], Woo Jong Cho[1], Il-Seung Youn[1], Han Myoung Lee[1], Muhammad Yousuf[1], Nisar Ahmed[3], Bahareh Shirinfar[3], Aaron J. Teator[1,4], Dominika N. Lastovickova[1,4], Lubna Rasheed[1], Myoung Soo Lah[1], Christopher W. Bielawski[1], and Kwang S. Kim[1]

[1]Department of Chemistry, Ulsan National Institute of Science and Technology (UNIST), Ulsan 44919, Korea.
[2]Department of Chemistry, Pohang University of Science and Technology, Pohang 790-784, Korea.
[3]School of Chemistry, University of Bristol, Bristol, BS8 1TS, UK.
[4]Department of Chemistry, The University of Texas at Austin, Austin, TX 78712, USA.

Correspondence and requests for materials should be addressed to K.S.K. (email: kimks@unist.ac.kr) or C.W.B. (email: bielawski@unist.ac.kr), or M.S.L. (email: mslah@unist.ac.kr).

CONTENTS

1. NMR Spectra
2. Crystallographic Information
3. Computation Results

4. NMR spectra

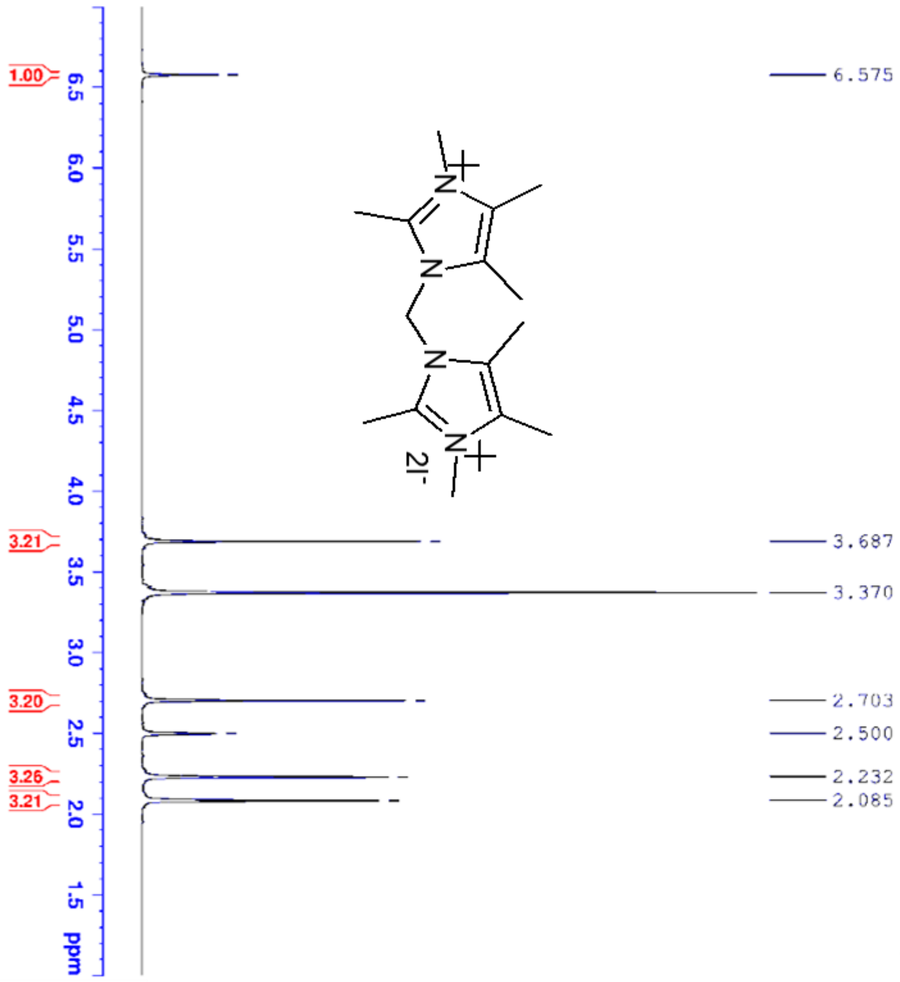

Figure S1-1. ^{1}H NMR spectrum of compound **[1](I)$_2$**in d_6-DMSO.

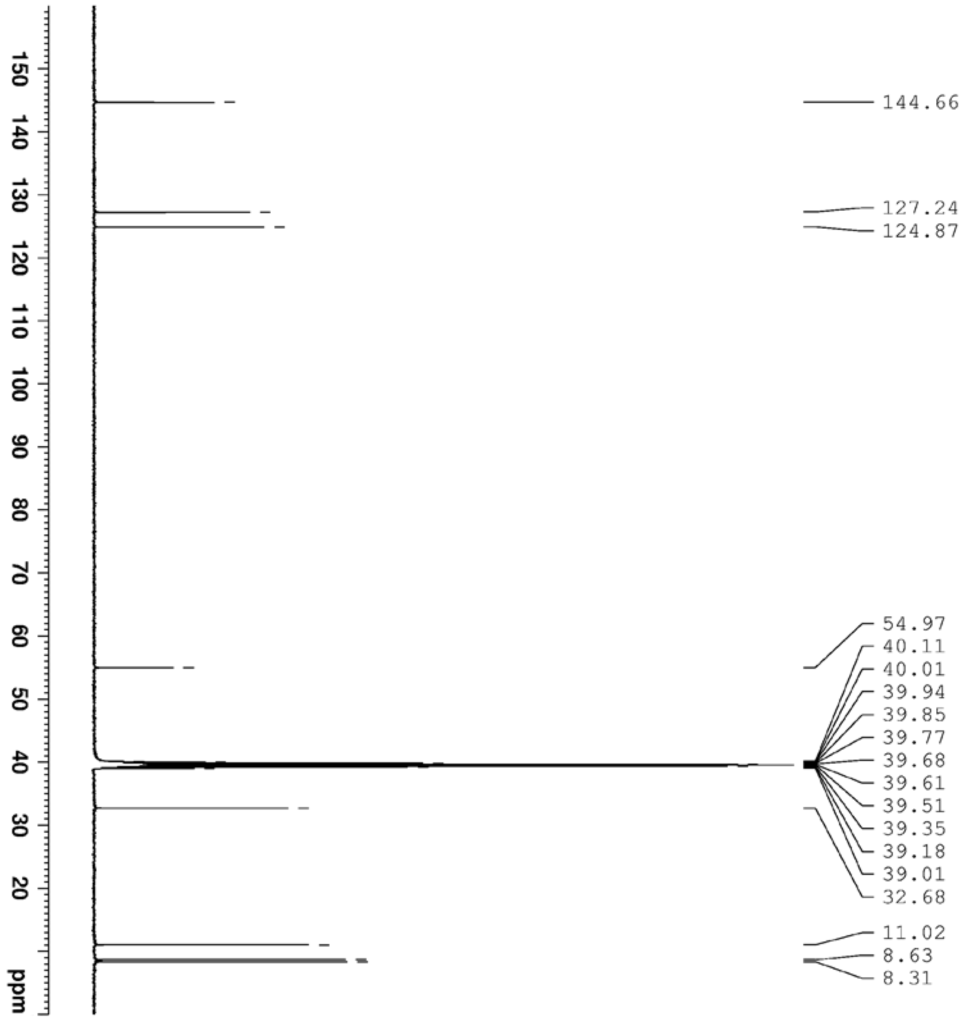

Figure S1-2. ^{13}C NMR spectrum of compound **[1](I)$_2$** in d_6-DMSO.

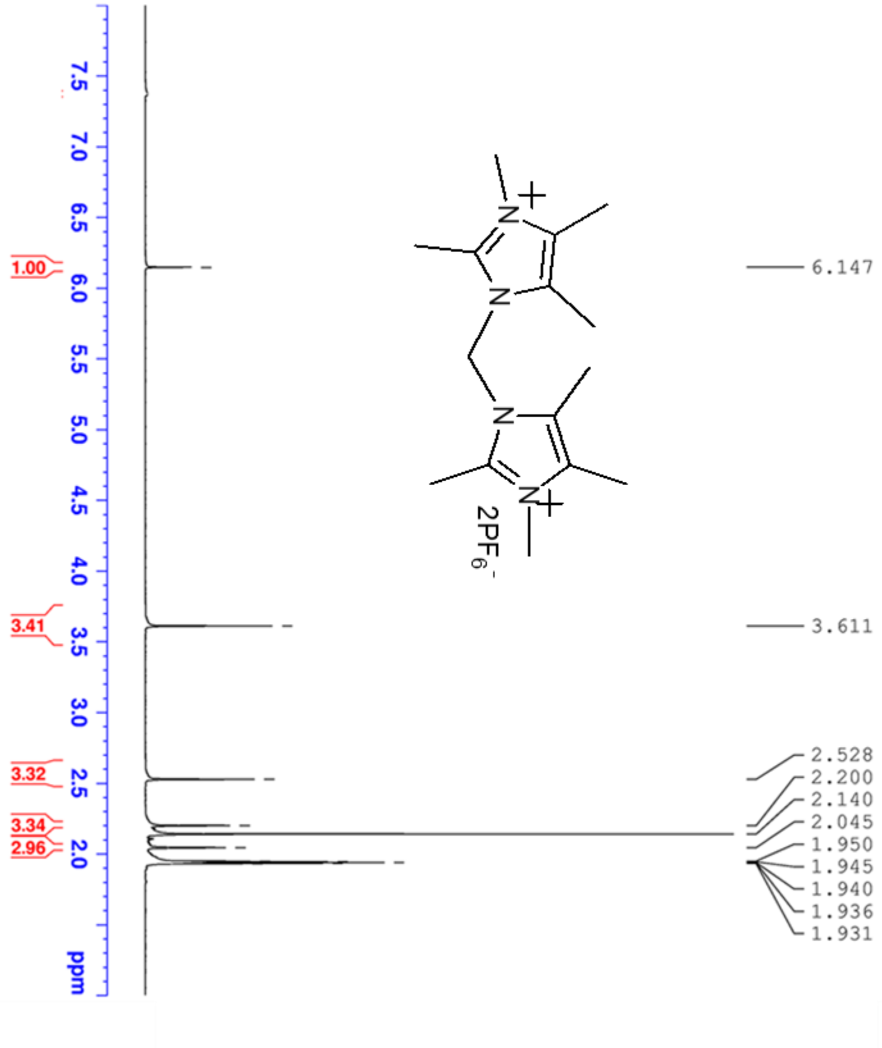

Figure S1-3. ^{1}H NMR spectrum of compound [**1**]$(PF_6)_2$ in CD_3CN.

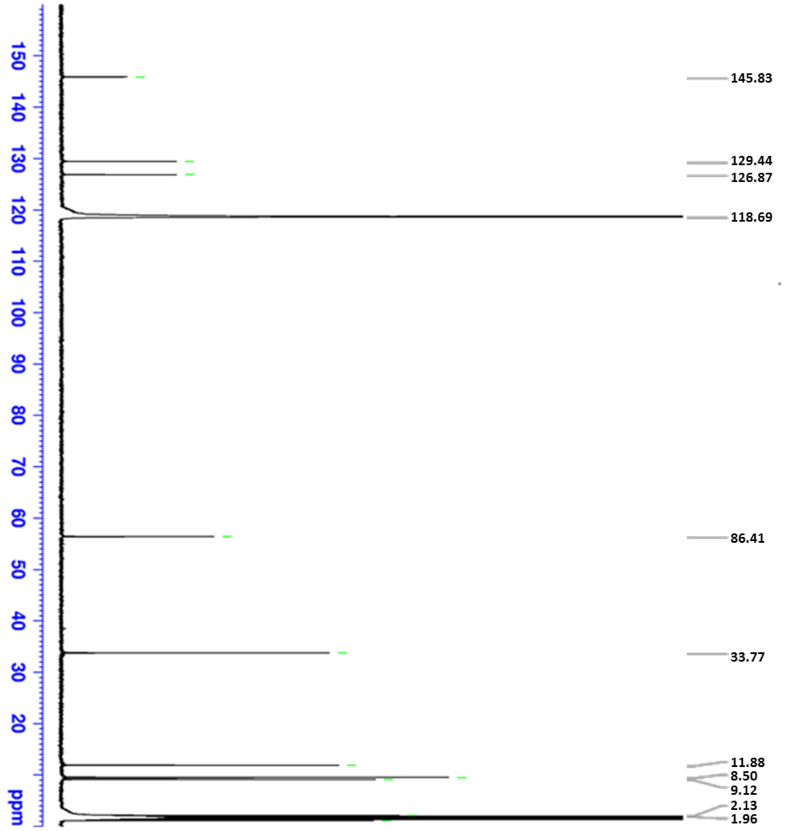

Figure S1-4. ^{13}C NMR spectrum of compound [**1**](PF_6)$_2$ in CD_3CN.

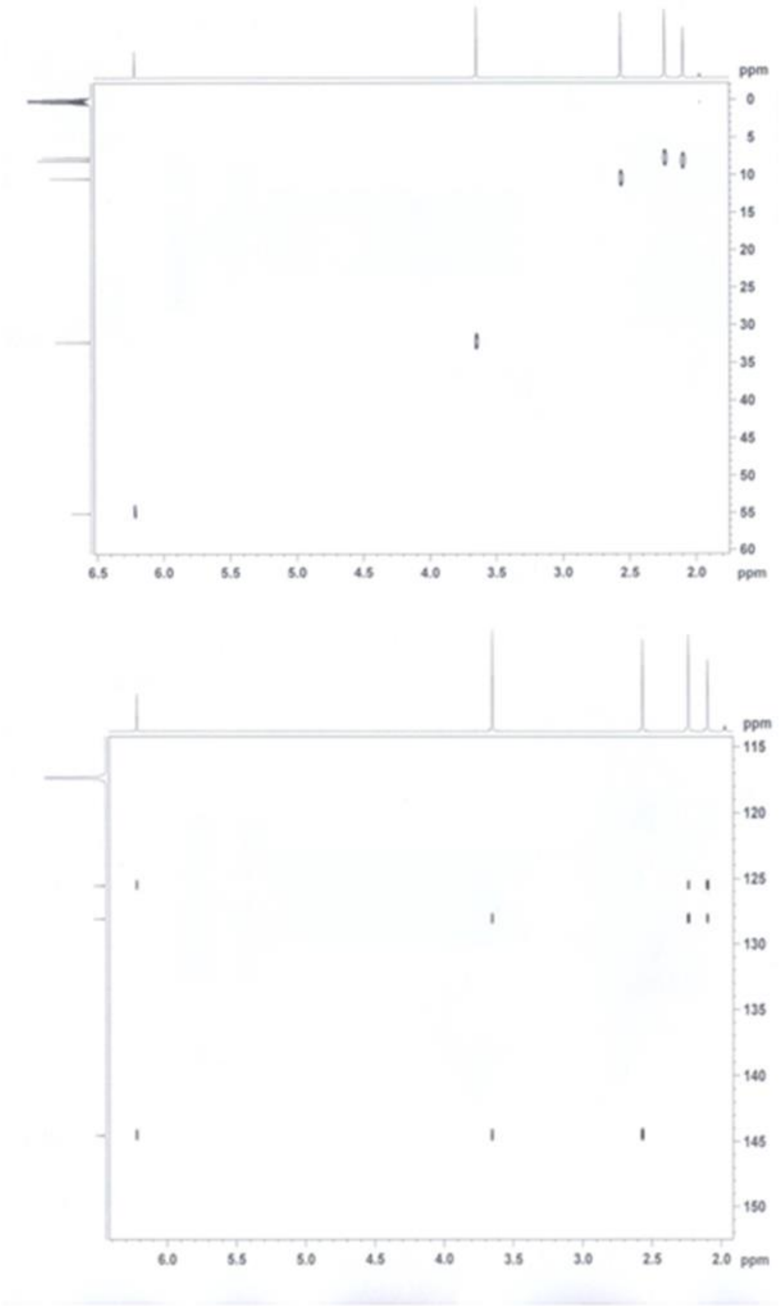

Figure S1-5. C-H COSY of compound [**1**](PF_6)$_2$ in CD_3CN.

5. Crystallographic Information

(1) Crystallographic data collection and refinement of the structure

Crystal [**1**]$(Cl)_2$ were coated with paratone oil and the diffraction data were measured at 173 K with Mo Kα radiation on an X-ray diffraction camera system using an imaging plate equipped with a graphite crystal incident beam monochromater. The RapidAuto software[1] was used for data collection and data processing. Structure was solved by direct method and refined by full-matrix least-squares calculation with the SHELXTL software package.[2]

For [**1**]$(Cl)_2$, one bis-imidazolium cation and two chloride anions are observed as an asymmetric unit. All non-hydrogen atoms are refined anisotropically; The hydrogen atoms of respective atom were found in the difference Fourier map and refined with isotropic displacement coefficients U(H) = 1.2U. Refinement of the structure converged at a final $R1 = 0.0406$ and $wR2 = 0.0801$ for 2333 reflections with $I > 2\sigma(I)$; $R1 = 0.1020$ and $wR2 = 0.1201$ for all 3842 reflections. The largest difference peak and hole were 0.569 and −0.579 e·Å^{-3}, respectively.

A summary of the crystals and some crystallography data are given in **Table S2-1**. 1062533 contain the supplementary crystallographic data for this paper. The data can be obtained free of charge at www.ccdc.cam.ac.uk/conts/retrieving.html or from the Cambridge Crystallographic Data Centre, 12, Union Road, Cambridge CB2 1EZ, UK.

1. Crystallographic Information

(1) Crystallographic data collection and refinement of the structure

Crystal [**1**]$(Cl)_2$ were coated with paratone oil and the diffraction data were measured at 173 K with Mo Kα radiation on an X-ray diffraction camera system using an imaging plate equipped with a graphite crystal incident beam monochromater. The RapidAuto software[3] was used for data collection and data processing. Structure was solved by direct method and refined by full-matrix least-squares calculation with the SHELXTL software package.[4]

For [**1**]$(Cl)_2$, one bis-imidazolium cation and two chloride anions are observed as an asymmetric unit. All non-hydrogen atoms are refined anisotropically; The hydrogen atoms of respective atom were found in the difference Fourier map and refined with isotropic displacement coefficients U(H) = 1.2U. Refinement of the structure converged at a final $R1 = 0.0406$ and $wR2 = 0.0801$ for 2333 reflections with $I > 2\sigma(I)$; $R1 = 0.1020$ and $wR2 = 0.1201$ for all 3842 reflections. The largest difference peak and hole were 0.569 and −0.579 $e{\cdot}Å^{-3}$, respectively.

A summary of the crystals and some crystallography data are given in **Table S2-1**. 1062533 contain the supplementary crystallographic data for this paper. The data can be obtained free of charge at www.ccdc.cam.ac.uk/conts/retrieving.html or from the Cambridge Crystallographic Data Centre, 12, Union Road, Cambridge CB2 1EZ, UK.

(2) Detailed crystallographic data of [**1**](Cl)$_2$

Table S2-1. Crystal data and structure refinement for [**1**](Cl)$_2$.

Empirical formula	$C_{15}H_{26}N_4Cl_2$	
Formula weight	333.30	
Temperature	173(2) K	
Wavelength	0.71073 Å	
Crystal system	Triclinic	
Space group	P-1	
Unit cell dimensions	a = 6.9352(14) Å	α = 89.68(3)°
	b = 10.227(2) Å	β = 87.52(3)°
	c = 12.049(2) Å	γ = 83.28(3)°
Volume	847.9(3) Å^3	
Z	2	
Density (calculated)	1.305 Mg/m^3	
Absorption coefficient	0.383 mm^{-1}	
F(000)	356	
Crystal size	0.150 x 0.150 x 0.140 mm^3	
Theta range for data collection	3.347 to 27.453°.	
Index ranges	–8<=h<=8, –13<=k<=12, –15<=l<=15	
Reflections collected	8401	
Independent reflections	3842 [R(int) = 0.0419]	
Completeness to theta = 25.242°	99.7 %	
Absorption correction	Semi-empirical from equivalents	
Max. and min. transmission	0.948 and 0.945	
Refinement method	Full-matrix least-squares on F^2	
Data / restraints / parameters	3842 / 0 / 268	
Goodness-of-fit on F^2	1.176	
Final R indices [I>2sigma(I)]	R1 = 0.0406, wR2 = 0.0801	
R indices (all data)	R1 = 0.1020, wR2 = 0.1201	
Extinction coefficient	n/a	
Largest diff. peak and hole	0.569 and –0.579 e·Å^{-3}	

Table S2-2. Atomic coordinates (x 10^4) and equivalent isotropic displacement parameters ($Å^2$x 10^3) for [**1**]$(Cl)_2$. U(eq) is defined as one third of the trace of the orthogonalized U^{ij} tensor.

	x	y	z	U(eq)
N(1)	6969(3)	7988(2)	1572(2)	17(1)
N(2)	5466(3)	7916(2)	36(2)	22(1)
N(3)	8016(3)	7065(2)	3351(2)	19(1)
N(4)	7588(3)	6511(2)	5075(2)	22(1)
C(1)	7121(4)	7491(3)	524(2)	20(1)
C(2)	4254(4)	8704(2)	768(2)	18(1)
C(3)	5176(4)	8748(3)	1728(2)	20(1)
C(4)	8792(5)	6695(3)	-47(3)	25(1)
C(5)	5005(6)	7549(4)	-1081(3)	38(1)
C(6)	2270(4)	9267(3)	485(3)	27(1)
C(7)	4477(5)	9384(3)	2790(3)	28(1)
C(8)	7912(4)	5708(3)	3368(2)	19(1)
C(9)	7644(4)	5369(3)	4446(2)	20(1)
C(10)	7839(4)	7539(3)	4408(2)	20(1)
C(11)	8075(5)	4893(3)	2347(3)	23(1)
C(12)	7469(5)	4059(3)	4957(3)	26(1)
C(13)	7236(5)	6626(4)	6284(3)	30(1)
C(14)	7922(5)	8890(3)	4806(3)	28(1)
C(15)	8507(4)	7837(3)	2373(2)	20(1)
Cl(1)	2923(1)	5975(1)	1816(1)	33(1)
Cl(2)	9163(1)	1227(1)	2693(1)	33(1)

Table S2-3. Bond lengths [Å] and angles [°] for [**1**]($Cl)_2$.

N(1)-C(1)	1.359(3)
N(1)-C(3)	1.393(3)
N(1)-C(15)	1.463(4)
N(2)-C(1)	1.337(4)
N(2)-C(2)	1.385(3)
N(2)-C(5)	1.459(4)
N(3)-C(10)	1.361(4)
N(3)-C(8)	1.398(3)
N(3)-C(15)	1.465(3)
N(4)-C(10)	1.343(3)
N(4)-C(9)	1.392(4)
N(4)-C(13)	1.469(4)
C(1)-C(4)	1.481(4)
C(2)-C(3)	1.350(4)
C(2)-C(6)	1.481(4)
C(3)-C(7)	1.475(4)
C(8)-C(9)	1.353(4)
C(8)-C(11)	1.482(4)
C(9)-C(12)	1.487(4)
C(10)-C(14)	1.473(4)
C(1)-N(1)-C(3)	109.0(2)
C(1)-N(1)-C(15)	125.3(2)
C(3)-N(1)-C(15)	125.5(2)
C(1)-N(2)-C(2)	109.7(2)
C(1)-N(2)-C(5)	124.4(3)
C(2)-N(2)-C(5)	125.9(3)
C(10)-N(3)-C(8)	109.5(2)
C(10)-N(3)-C(15)	124.4(2)
C(8)-N(3)-C(15)	125.6(2)
C(10)-N(4)-C(9)	109.9(2)
C(10)-N(4)-C(13)	123.6(3)
C(9)-N(4)-C(13)	126.5(2)

N(2)-C(1)-N(1)	107.0(2)
N(2)-C(1)-C(4)	124.0(3)
N(1)-C(1)-C(4)	128.9(3)
C(3)-C(2)-N(2)	107.5(2)
C(3)-C(2)-C(6)	130.1(3)
N(2)-C(2)-C(6)	122.3(3)
C(2)-C(3)-N(1)	106.8(2)
C(2)-C(3)-C(7)	129.3(3)
N(1)-C(3)-C(7)	123.8(3)
C(9)-C(8)-N(3)	106.6(2)
C(9)-C(8)-C(11)	130.5(3)
N(3)-C(8)-C(11)	122.9(2)
C(8)-C(9)-N(4)	107.4(2)
C(8)-C(9)-C(12)	130.2(3)
N(4)-C(9)-C(12)	122.4(3)
N(4)-C(10)-N(3)	106.6(2)
N(4)-C(10)-C(14)	124.2(3)
N(3)-C(10)-C(14)	129.2(3)
N(1)-C(15)-N(3)	113.0(2)

Symmetry transformations used to generate equivalent atoms:

Table S2-4. Anisotropic displacement parameters ($Å^2$x 10^3) for [**1**]$(Cl)_2$. The anisotropic displacement factor exponent takes the form: $-2p^2[h^2 a^{*2}U^{11} + ... + 2 h k a^* b^* U^{12}]$

	U^{11}	U^{22}	U^{33}	U^{23}	U^{13}	U^{12}
N(1)	21(1)	15(1)	15(1)	0(1)	-4(1)	1(1)
N(2)	28(1)	22(1)	16(1)	-2(1)	-6(1)	-1(1)
N(3)	22(1)	18(1)	16(1)	0(1)	-2(1)	0(1)
N(4)	24(1)	26(1)	16(1)	0(1)	-4(1)	-1(1)
C(1)	25(2)	18(1)	17(2)	-1(1)	-3(1)	-2(1)
C(2)	18(1)	15(1)	21(2)	3(1)	-3(1)	-2(1)
C(3)	23(2)	18(1)	20(2)	1(1)	-2(1)	-1(1)
C(4)	30(2)	26(2)	19(2)	-3(1)	-3(1)	1(1)
C(5)	49(2)	32(2)	30(2)	-9(2)	-15(2)	10(2)
C(6)	26(2)	27(2)	28(2)	1(1)	-5(1)	-1(1)
C(7)	28(2)	31(2)	24(2)	-3(1)	-4(1)	5(1)
C(8)	16(1)	19(1)	22(2)	0(1)	-4(1)	1(1)
C(9)	17(1)	22(2)	21(2)	-1(1)	-4(1)	-2(1)
C(10)	23(1)	22(2)	16(1)	0(1)	-3(1)	1(1)
C(11)	32(2)	17(2)	21(2)	-2(1)	-5(1)	-2(1)
C(12)	24(2)	25(2)	30(2)	5(1)	0(1)	-3(1)
C(13)	39(2)	35(2)	15(2)	1(1)	-3(1)	-6(2)
C(14)	41(2)	24(2)	20(2)	-2(1)	-6(1)	-3(1)
C(15)	21(1)	19(2)	19(2)	2(1)	-2(1)	-3(1)
Cl(1)	27(1)	29(1)	44(1)	11(1)	-3(1)	-2(1)
Cl(2)	42(1)	23(1)	34(1)	1(1)	-14(1)	-3(1)

Table S2-5. Hydrogen coordinates (x 10^4) and isotropic displacement parameters ($Å^2$x 10^3) for [**1**]$(Cl)_2$.

	x	y	z	U(eq)
H(4A)	9880(50)	6440(30)	420(30)	30
H(4B)	8370(40)	5940(30)	-310(30)	30
H(4C)	9270(40)	7170(30)	-680(30)	30
H(5A)	5360(50)	6680(40)	-1220(30)	45
H(5B)	5950(50)	7980(30)	-1650(30)	45
H(5C)	3900(60)	7920(40)	-1250(30)	45
H(6A)	1490(50)	8610(30)	420(30)	32
H(6B)	2250(40)	9740(30)	-230(30)	32
H(6C)	1690(50)	9870(30)	1060(30)	32
H(7A)	5350(50)	9970(30)	3060(30)	34
H(7B)	4370(50)	8790(30)	3400(30)	34
H(7C)	3240(50)	9890(30)	2710(30)	34
H(11A)	8090(40)	3980(30)	2530(30)	28
H(11B)	9310(50)	5000(30)	1920(30)	28
H(11C)	7050(50)	5130(30)	1910(30)	28
H(12A)	8320(50)	3840(30)	5530(30)	32
H(12B)	7760(40)	3370(30)	4420(30)	32
H(12C)	6220(50)	4010(30)	5340(30)	32
H(13A)	8120(50)	7100(30)	6610(30)	36
H(13B)	7300(50)	5780(30)	6630(30)	36
H(13C)	5960(50)	7060(30)	6460(30)	36
H(14A)	6570(50)	9270(30)	5130(30)	34
H(14B)	8780(50)	8840(30)	5410(30)	34
H(14C)	8290(50)	9420(30)	4270(30)	34
H(15A)	9740(40)	7370(30)	2030(20)	23
H(15B)	8700(40)	8700(30)	2590(20)	23

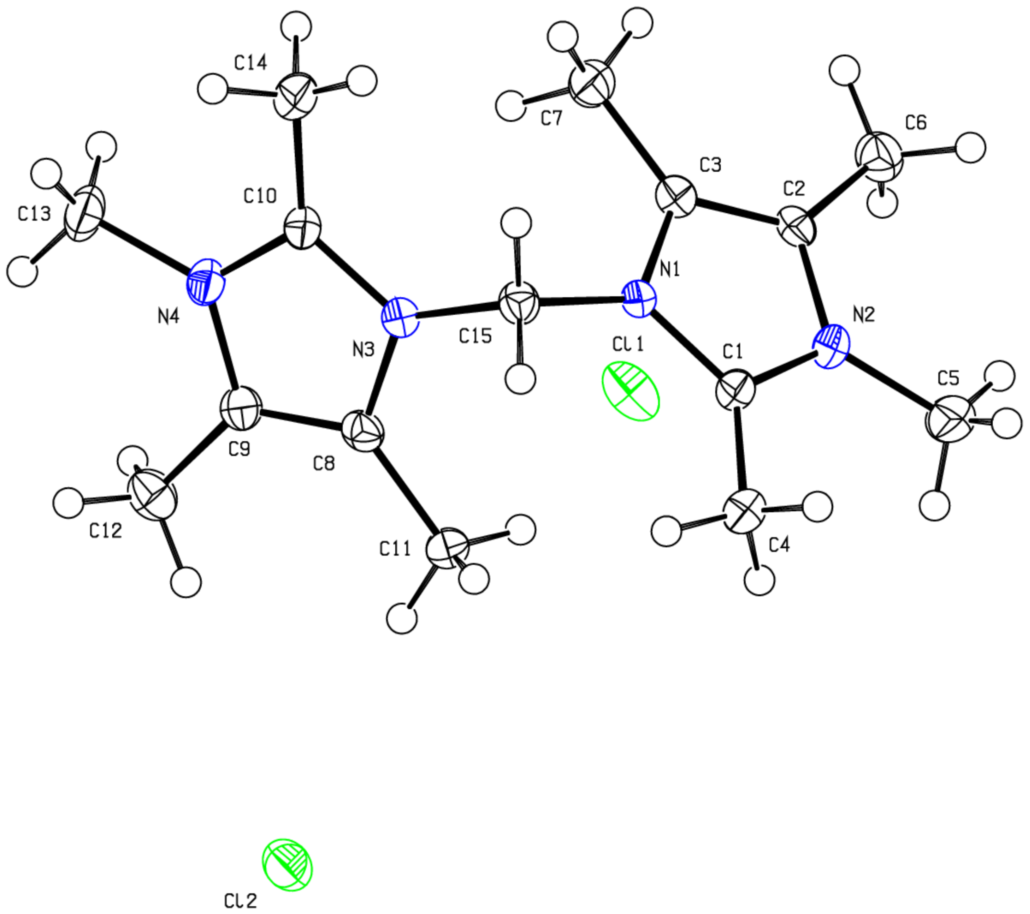

Figure S2-1. Crystal structures of [**1**]Cl_2 drawn using ORTEP (Green: Cl, Blue: N, White with stripe: C, White without stripe: H).

3. Computational Results

3.1 Orbital overlaps between Cl^- and the H-C_{ali} moiety in the HOMOs.

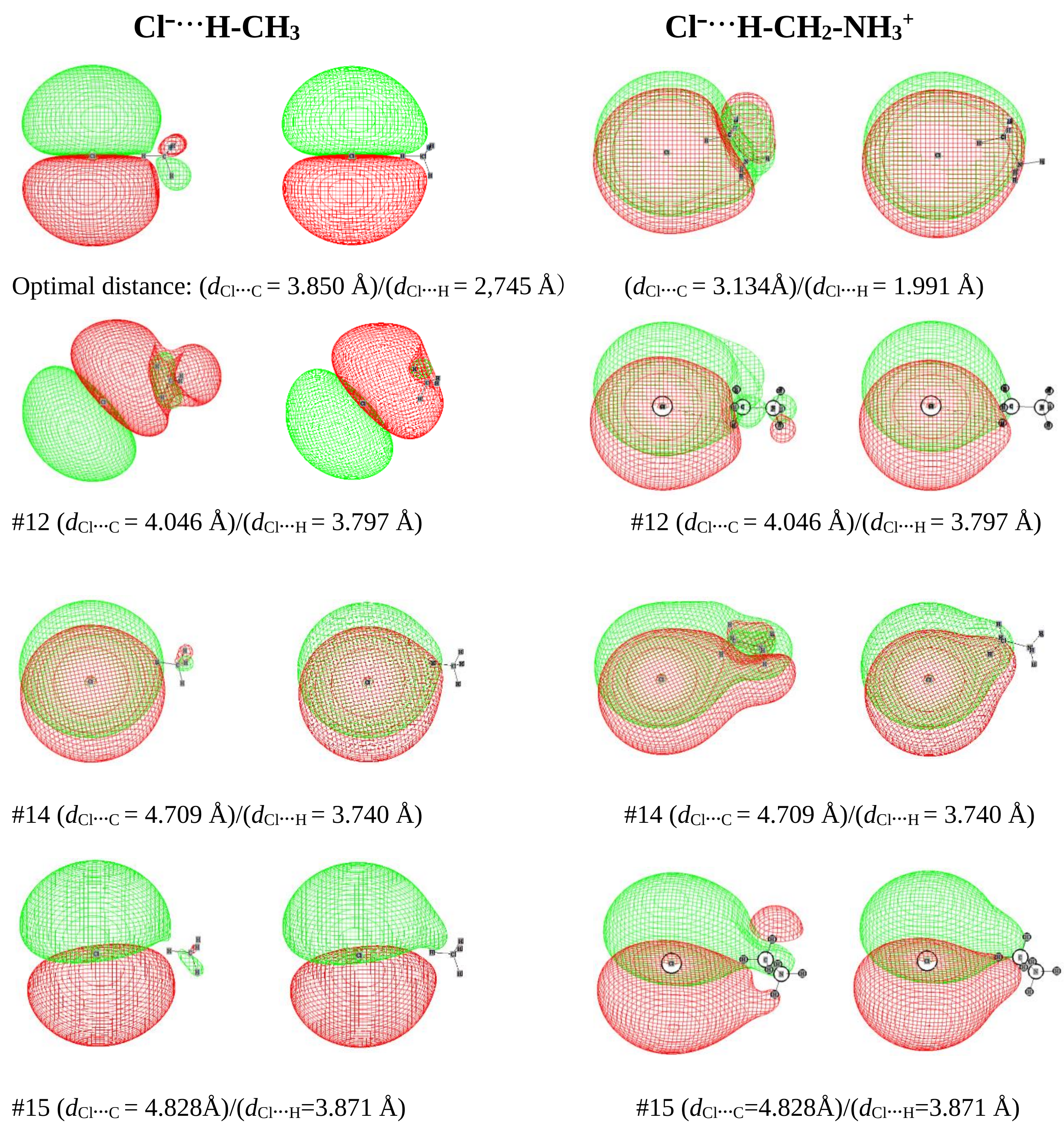

Figure S3-1. HOMO maps (left: orbitals from all atoms, right: orbitals only from Cl^-···H atoms) for Cl^-···H Hydrogen-bonding-like interactions in the Cl^-···H-CH_3 and Cl^-···H-CH_2-NH_3^+ molecular systems using the geometries of crystal structure of **[1]**(Cl_2), where the non H atom has been replaced by H using a proper C-H/N-H bond length. Though the overlaps are small, they shows bonding-type overlaps, implying a minimal feature of covalent bonding still even at somewhat long Cl...H distances. The structures optimized at the B97D/cc-

PVDZ level (which show similar HOMO maps to most cases of H atom#1-11, and 13 in Table S3-4) and the structures for the cases of H atoms # 12, 14, and 15 (which are somewhat longer than the typical H-bond distance) are considered in this figure. The calculations were performed using GAUSSIAN09 suite of programs[3].

3.2 DFT-SAPT results

The symmetry adapted perturbation theory calculations using density functional theory (DFT-SAPT) were performed for component-wise analyses of the interaction between Cl^- and methyl group: E_{es} (electrostatic energy), E_{ind}^* (effective induction energy including the induction-induced exchange energy; $E_{ind}^* = E_{ind} + E_{ind\text{-}exch}$), E_{disp}^* (effective dispersion energy including the dispersion-induced exchange energy; $E_{disp}^* = E_{disp} + E_{disp\text{-}exch}$), E_{exch}^* (effective exchange repulsion energy with the induction-induced and dispersion-induced exchange energies excluded; $E_{exch}^* = E_{exch} - (E_{ind\text{-}exch} + E_{disp\text{-}exch})$), and E_{tot} (total interaction energy) with δH, contribution of third and higher order terms at the uncorrelated level. We used the PBE0AC/aug-cc-pVDZ (aVDZ) xc functional and basis set with the ALDA xc kernel. All the SAPT computations were performed with Molpro package.[4,5] The model systems, $CH_4\cdots Cl^-$ and $NH_3CH_3^+\cdots Cl^-$ were chosen from the crystal structures in which only the positions of hydrogen atoms were re-optimized. Here in this study, the H-bonding-like interactions are described in terms of the $Cl^-\cdots H_x$ distances (H_x: hydrogen atom directly interacting with Cl^-) (Tables S3-1 and S3-3).

For investigating the angle-dependence of long range H-bonding-like interactions, we also conducted calculations for $CH_4\cdots Cl^-$ with different $Cl^-\cdots H_x$-C angles (θ). For each system, only the interaction angle was fixed, while other parameters were optimized at the B97D/aVDZ level. The $Cl^-\cdots H_x$ and H_x-C distances are shown in Table S3-2.

Table S3-1. DFT-SAPT (PBE0AC/aug-cc-pVDZ) calculation results for the $CH_4\cdots Cl^-$ model systems with different $Cl^-\cdots H_x$ distances (kcal/mol).

#H-atom	(optimal)	#14	#12	#15
$d_{Cl\cdots C}$=4.828Å	3.850	4.709	4.046	4.828
$d_{Cl\cdots H}$=3.871Å	2.705	3.740/3.664	3.797/3.719	3.871/3.733
angle(θ)	180°	153°	100°	177°
E_{tot}	-3.51	-2.25	-2.13	-2.33
E_{es}	-2.40	-0.23	-0.09	-0.36
E_{ind}^*	-2.37	-1.01	-1.41	-0.95
E_{disp}^*	-1.68	-0.42	-0.87	-0.36
E_{exch}^*	3.81	0.29	1.11	0.22

Table S3-2. Interaction angle θ ($Cl^-\cdots H_x$-C) vs. $d_{Cl^-\cdots Hx/Hx\text{-}C}$ dependence for the $Cl^-\cdots HCH_3$ model system at the B97D/aVDZ level, showing some of H-bonding characters.

∠$Cl^-\cdots H_x$-C	$Cl^-\cdots H_x$ (Å)	H_x-C (Å)	BE
180° (opt)	2.705	1.108	-3.06
160	2.759	1.107	-2.85

140	2.879	1.105	-2.35
120	3.145	1.102	-1.88
100	3.500	1.102	-2.00

Table S3-3. DFT-SAPT energy decomposition (in kcal/mol) for the $NH_3CH_3^+\cdots Cl^-$ model system for selected H atoms of the following $Cl^-\cdots H_x$ distances.

Distance (Å)	1.991	3.269	3.699	3.723
E_{tot}	-96.40	-120.11	-78.97	-68.41
E_{es}	-100.06	-107.47	-72.21	-63.88
E_{ind}^*	-9.81	-12.49	-2.83	-1.99
E_{disp}^*	-5.44	-3.99	-0.54	-0.38
E_{exch}^*	29.79	16.23	0.44	0.27

3.3 QTAIM results

The quantum theory of atoms in molecules (QTAIM) analysis was used to determine the presence of bond critical points ((3,–1) BCPs) of the intramolecular H-bonding-like interactions in $CH_4\cdots Cl^-$ and $NH_3CH_3^+\cdots Cl^-$ model systems as well as the crystal structure model of **[1]**(Cl_2). According to AIM theory[6] the presence of interaction between X and Y atoms must correspond to the existence of a bond path between X and Y atoms containing the BCP, in the topological analysis of the electron density distribution. Based on the IUPAC definition for the hydrogen bond "*the electron density topology of hydrogen-bonded systems usually shows a bond path connecting H and Y and a (3,–1) bond critical point between H and Y*"[7], the Laplacian of electron density is related to the bond interaction energy by a local expression of the viral theorem:[8,9]

$$\frac{\hbar^2}{4m}\nabla^2\rho(r) = 2\,G(r) + V(r)$$

where *G(r)* is the electronic kinetic energy density and *V(r)* is the electronic potential energy density. A negative $\nabla^2\rho$ (r) shows the excess potential energy at the BCP which means that the electronic charge is contracted between two nuclei (covalent interaction), while a positive $\nabla^2\rho$ (r) reveals that the kinetic energy contribution is greater than the potential energy (closed-shell electrostatic interaction). The sign of electronic energy *H(r)* on the Hamiltonian at BCP determines whether the accumulation of charge at a given point is stabilizing (*H(r)* < 0) or destabilizing (*H(r)* > 0). The nature of hydrogen bond can be evaluated by means of the $\frac{-G(r)}{V(r)}$ ratio. When $\frac{-G(r)}{V(r)} > 1$, the hydrogen bond has non-covalent character while for the $0.5 < \frac{-G(r)}{V(r)} < 1$ ratio, the hydrogen bond has partly covalent character.[8,9] The relating distances and angles and QTAIM topological parameters for $Cl^-\cdots H_x$ interaction of the CH_4-Cl^- and $NH_3CH_3^+\cdots Cl$ models are shown in Table S3-4. All the QTAIM computations were performed with AIMALL package[10] with .wfx and .fchk files generated in Gaussian[3] used as the input. As can be seen from Table S3-4 results, all of $Cl^-\cdots H_x$ H-bonding-like interactions in models systems are non-covalent (from the $\frac{-G(r)}{V(r)}$ ratio) with closed-shell and show the electrostatic nature ($\nabla^2\rho$ (r) >0). In the **[1]**Cl_2 crystal structure, QTAIM results confirm

(Table S3-5 and Figure S3-2) fifteen interaction sites of the chloride anion through $Cl^{-}\cdots H_x$ H-bonding-like interactions. These indicate electrostatic character ($\nabla^2\rho$ (r) >0) and most of their topological parameters are consistent with the range of conventional hydrogen bonds (ρ(r)= 0.002–0.040 a.u and $\nabla^2\rho$ (r)= 0.024–0.139 a. u) suggested by Koch and Popelier.[11]

Koch and Popelier topological parameters (ρ(r) = 0.002–0.035 a.u. and $\nabla^2\rho(r)$ = 0.024–0.139 a.u in BCPs) were developed in the context of a limited set of H-bond types (such as C–H…O, RCN…HF and RCN…HCl). A number of additional criteria (not relating to BCP) were also proposed by them. In imidazolium cation the H-bond donor is the C–H unit (C–H on the aromatic ring or a group in the alkyl chains) which can be formed in an array of H-bonds ($C\text{-}H^{+}\ldots X^{-}$ as doubly ionic H-bond) with different strength and type with halide species in ionic liquids. Doubly ionic H-bonds occur when an H-bond forms between a cation and anion, and are a key feature of ionic liquids. However, there are very few studies that address topological parameters for the doubly ionic H-bond[12,13] and could not be practical in this regard. A common parameter in the description of H-bonds is the extent of mutual interpenetration of the closed shell electron clouds which represented by the difference between sum of the van der Waals radii of H and Y and the H…Y distance. Moreover, the IUPAC description of H-bonding indicates that H and Y should be connected by a (3, -1) bond critical point. Therefore, we tried to characterize C-H...Cl^{-} hydrogen bonds by considering the H-bond distances and the sum of r^{vdW} radii of Cl^{-} and H. We clearly stated that eleven binding sites are involved in H-bonding because these H-bond distances are smaller or practically equivalent to the sum of r^{vdW} radii of Cl^{-} and H or C-H..Cl^{-}, while two binding sites belong to the same bi-dentate H-bonding, and two remaining binding sites are simply H-bond-like interaction sites.

The computed electron density (ρ(r)), the ratio of Laplacian of electron density ($\nabla^2\rho(r)$) or kinetic energy density (G(r)) to potential energy density (V(r)), and the electronic energy density (H(r)) at C-H..Cl^{-} H-bonds BCPs for bis-imidazolium tetraalkylammoinium and crystal structure of **[1]**$(Cl)_2$ are summarized in Tables S3-4 and S3-5 in the Supporting Information. The value of the electron density ρ(r) and the sign of its $\nabla^2\rho(r)$ at the BCP are used to characterize H-bonds. The electron densities are distributed in the range of ρ(r) = 0.001-0.014 a.u for bis-imidazolium, ρ(r) = 0.002-0.015 a.u for tetraalkylammoinium and ρ(r) = 0.001-0.011 a.u for crystal structure of **[1]**$(Cl)_2$, respectively (the typical value of ρ(r) is 0.01 a.u. for H-bonds) which reflect the smooth transition from weak hydrogen bond to moderate (classical) and strong hydrogen bond. The electron density (ρ(r)) is usually used to describe the strength of a bond. The small value of electron density (in weak closed shell bonds such as H-bonds, noble-gas dimers, and ionic systems) indicates that the H-bond is primarily ionic (electrostatic) in character while for a covalent bond, ρ(r) in BCP is typically greater than 0.1 a.u. It is worth to mention that the exponential relationship between of the electron density ρ(r) and C-H..Cl^{-} H-bond distances, same as in traditional H-bonds, are also observed in these models. The sign of Laplacian of electron density $\nabla^2\rho(r)$ are used to characterize a bond. For H-bonds the Laplacian is usually small and positive (see Tables S3-4 and S3-5 in the Supporting Information). Meanwhile, the combined curvature at the BCP is very flat with closed shell (electrostatic) type of bonding[13]. On the other hand, negative and positive values of H(r) for H-bonds imply the covalent and electrostatic nature of the corresponding H-bonds, respectively. The positive values of H(r) for C-H..Cl^{-} BCP in our models has been

associated with a dominant electrostatic contribution (see Tables S3-4 and. S3-5 in the Supporting Information). Therefore, as can be seen from Tables S3-4 and S3-5, all of $Cl^{-}\cdots H_x$ H-bonding-like interactions in models systems are non-covalent (from the $\frac{-G(r)}{V(r)}$ ratio) with closed-shell and show the electrostatic nature ($\nabla^2\rho$ (r) >0).

To understand non-covalent interactions in more detail, the Laplacian is often decomposed into a sum of contributions (eigenvalues λ_i of the electron-density Hessian (second derivative) matrix as λ_1, λ_2 and λ_3). Analysis of the sign of λ_2 enables to distinguish different types of non-covalent interactions such as steric clashes (positive λ_2), attractive H-bonding (negative λ_2) and vdW interactions with a negligible density overlap. QTIAM topological parameters and the λ_2 value for the crystal structure of **[1]**$(Cl)_2$ are given in Table S3-5. Negative sign of λ_2 demonstrates that all of C-H..Cl^{-} interactions in **[1]**$(Cl)_2$ crystal are attractive. We also used the sign of this eigenvalue to characterize the nature of interactions based on the iso-surface coloring in the NCIPLOT program. NCI surfaces are colored via the strength of the interaction, blue (strong attractive, with electrostatic contribution), green (weak, with VdW contribution) and red (repulsive). The iso-surface of sign (λ_2) × ρ defined by Yang et al.[14] is displayed for the **[1]**$(Cl)_2$ crystal in Figure S3-2. In this figure, the strong intermolecular interactions appear as round blue domains (inset (**a**), realistic $Cl^{-}\cdots H_x$ H-bonds, $d_{Cl\cdots Hx} \leq 3.15$ Å) and weak H-bonding-like interactions with vdW contribution as green round domains (inset (**b**), $d_{Cl\cdots Hx} > 3.15$ Å). Therefore, the binding of chloride anion is favored by combination of short and long ranged specific H-bonding-like interactions (inclusion complex) in cooperative manner through the space in the cavity of crystal structure.

Table S3-4. Topological parameters for $CH_4\cdots Cl^{-}$ model systems with different $Cl^{-}\cdots H_x$ distances. Electron densities (ρ (r), e/a.u[3]), their Laplacians ($\nabla^2\rho$ (r), e/a.u[5]), electronic energy densities H(r) and the -G(r)/V(r) ratios at the $Cl^{-}\cdots H_x$ BCPs are calculated at the B97D/aVDZ level.

	[**a**] bis-imidazolium						[**b**] tetraalkylammoinium					
	d_{Cl^-H}	θ	ρ (r)	$\nabla^2\rho$ (r)	H (r)	-G(r)/V(r)	d_{Cl^-H}	θ	ρ (r)	$\nabla^2\rho$ (r)	H (r)	-G(r)/V(r)
1	2.646	167	0.0140	0.0354	0.000705	1.09	2.711	153	0.0149	0.0320	0.000302	1.04
2	2.743	175	0.0132	0.0330	0.000671	1.10	2.750	152	0.0141	0.0294	0.000273	1.04
3	2.857	156	0.0125	0.0308	0.000645	1.10	2.670	168	0.0133	0.0274	0.000256	1.04
4	2.743	173	0.0116	0.0288	0.000638	1.11	2.721	158	0.0125	0.0256	0.000263	1.04
5	2.834	163	0.0113	0.0294	0.000724	1.12	2.678	170	0.0113	0.0294	0.000724	1.12
6	2.844	171	0.0111	0.0279	0.000656	1.12	2.719	159	0.0119	0.0250	0.000302	1.05
7	2.834	165	0.0104	0.0260	0.000643	1.12	2.771	153	0.0112	0.0233	0.000301	1.06
8	3.087	148	0.0079	0.0198	0.000607	1.16	2.799	154	0.0085	0.0177	0.000332	1.09
9	2.994	172	0.0078	0.0174	0.000595	1.19	2.830	150	0.0068	0.0174	0.000595	1.19
10	3.075	174	0.0063	0.0161	0.000582	1.20	2.856	151	0.0063	0.0161	0.000582	1.20
11	3.355	129	0.0053	0.0167	0.000626	1.21	2.840	175	0.0070	0.0153	0.00043	1.15
12	3.797	100	0.0038	0.0107	0.000569	1.37	3.119	145	0.0053	0.0108	0.000553	1.35
13	3.145	179	0.0068	0.0154	0.000381	1.65	3.471	131	0.0021	0.0049	0.000342	1.62
14	3.740	153	0.0033	0.0095	0.000548	1.43	3.717	134	0.0026	0.0055	0.000302	1.56
15	3.871	177	0.0040	0.0044	0.000645	1.09						

Table S3-5. QTAIM topological parameters for the $Cl^{-}\cdots H$ H-bonding-like interactions in the crystal structure of **[1]**$(Cl)_2$ at the B97D/aVDZ level.

#atom	$d_{Cl\cdots C}$	$d_{Cl^{-}H}$ (Å)	θ	ρ (r)	$\nabla^2\rho$ (r)	H (r)	λ_2
1	3.574	2.646	167	0.0118	0.119	0.000519	-0.00967
2	3.628	2.743	175	0.0097	0.106	0.000646	-0.0078
3	3.648	2.857	156	0.0077	0.086	0.000654	-0.00598
4	3.695	2.743	173	0.0096	0.104	0.000648	-0.00759
5	3.761	2.834	163	0.0083	0.089	0.000602	-0.00629
6	3.763	2.844	171	0.008	0.087	0.000629	-0.00634
7	3.78	2.834	165	0.0082	0.089	0.000586	-0.00639
8	3.94	3.087	148	0.0054	0.062	0.000602	-0.022719
9	3.944	2.994	172	0.0062	0.067	0.000532	-0.02559
10	4.028	3.075	174	0.0053	0.058	0.000508	-0.00381
11	4.046	3.355	129	0.0033	0.042	0.000588	-0.00203
12	4.046	3.797	100	0.0015	0.02	0.000397	-0.00658
13	4.114	3.145	179	0.0046	0.051	0.000512	-0.00323
14	4.709	3.74	153	0.0017	0.022	0.000316	-0.0062
15	4.828	3.871	177	0.0012	0.016	0.000205	-0.00035

3.4 Plot of noncovalent interaction (NCI) regions (NCIPLOT)

The NCI regions[14,15] can provide the information about the extent of the interaction (with non-bonded attractive or repulsive interactions) in real space. The NCI surface for intermolecular interactions around Cl^{-} in crystal structure of **[1]**$(Cl)_2$ is shown in Figure S3-2. In the figure, the strong intermolecular interactions appear as round blue domains (inset (**a**), realistic $Cl^{-}\cdots H_x$ H-bonds, $d_{Cl\cdots Hx} \leq 3.145$ Å) and weak H-bonding-like interactions as green round domains (inset (**b**)). Therefore, the binding of chloride anion is favored by combination of specific H-bonding-like interactions. The NCI analysis clearly highlights how the Cl^{-}·fits and stabilizes by combination of short and long ranged interaction through the space in the cavity of crystal structure (Figure S3-2).

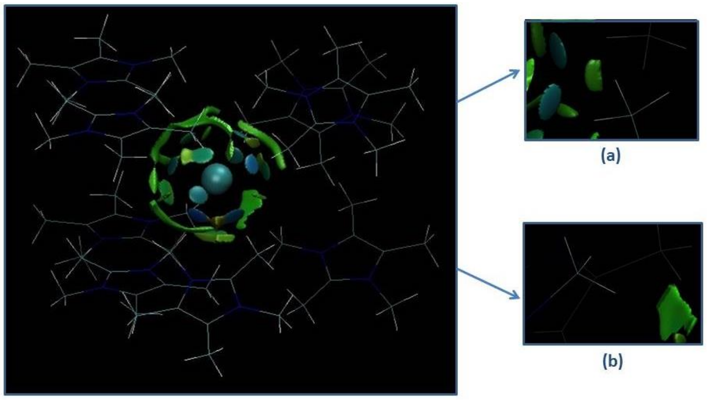

Figure S3-2. NCI surface for intermolecular interactions around Cl^{-} in crystal structure of **[1]**$(Cl)_2$. Specific interactions are enlarged in the insets ($d_{Cl\cdots Hx} \leq 3.145$ Å (**a**) and $d_{Cl\cdots Hx} > 3.145$ Å (**b**)). The gradient cutoff is s = 0.30 au, and the color scale is $-0.03 < \rho < 0.03$ au.

[1] Rapid Auto software, R-Axis series, Cat. No. 9220B101, Rigaku Corporation.
[2] SHELX program: Sheldrick, G. M. A short history of SHELX. *Acta Crystallogr Sect A* **64**, 112 (2008).
[3] Frisch, M. J. *et al. Gaussian 09, Revision A.02.* Gaussian, Inc., Wallingford CT, (2009).
[4] Werner, H.-J. *et al*. MOLPRO, version 2010.1, a package of *ab initio* programs.
[5] Bukowski, R. et al. sapt2012: An Ab Initio Program for Symmetry-Adapted Perturbation Theory Calculations of Intermolecular Interaction Energies. Sequential and parallel versions. User's Guide. Revision SAPT2012.2. (2013) http://www.physics.udel.edu/~szalewic/SAPT/manual.html. Date of access:01/03/2014..
[6] Bader, R. F. W. Atoms in Molecules. A Quantum Theory, Clarendon, Oxford, UK, 1990.
[7] Arunan, E. *et al*. Definition of the hydrogen bond (IUPAC Recommendations 2011). *Pure Appl Chem* **83**, 1637–1641 (2011).
[8] Popelier, P. L. A. Characterization of a dihydrogen bond on the basis of the electron density. *J Phys Chem A* **102**, 1873-1878 (1998)
[9]. Popelier, P. L. A. Atoms in molecules. An introduction. Prentice Hall, Harlow, 2000.
[10] AIMAll (Version 15.09.27), Todd, A.; Keith, T. K. Gristmill Software, Overland Park K. S., USA, 2015 (aim.tkgristmill.com)
[11] Koch, U., & Popelier, P. L. A. Characterization of C-H-O hydrogen bonds on the basis of the charge density. *J Phys Chem* **99**, 9747-9754 (1995).
[12] Matthews, R. P. Welton, T & Hunt, P. A. *Phys Chem Chem Phys* **17,** 14437-14453 (2015)
[13] Hunt, P. A. Ashworth, C. R. & Matthews, R. P. Hydrogen bonding in ionic liquids. *Chem Soc Rev* **44**, 1257-1288 (2015).
[14] Johnson, E. R., Keinan, S., Mori-Sánchez, P., Contreras-García, J., Cohen, A. J., Yang, W. Revealing noncovalent interactions. *J. Am. Chem. Soc*. **132**, 6498–6506 (2010).
[15] Contreras-García, *et al*. NCIPLOT: A program for plotting noncovalent interaction regions. *J Chem Theory Comput* **7**, 625-632 (2011).

[3] Rapid Auto software, R-Axis series, Cat. No. 9220B101, Rigaku Corporation.
[4] SHELX program: Sheldrick, G. M. A short history of SHELX. *Acta Crystallogr. Sect. A*. **64**, 112 (2008).
[3] Frisch, M. J. *et al. Gaussian 09, Revision A.02.* Gaussian, Inc., Wallingford CT, (2009).
[4] Werner, H.-J. *et al*. MOLPRO, version 2010.1, a package of *ab initio* programs.
[5] Bukowski, R. et al. sapt2012: An Ab Initio Program for Symmetry-Adapted Perturbation Theory Calculations of Intermolecular Interaction Energies. Sequential and parallel versions. User's Guide. Revision SAPT2012.2. (2013) http://www.physics.udel.edu/~szalewic/SAPT/manual.html. Date of access:01/03/2014..
[6] Bader, R. F. W. Atoms in Molecules. A Quantum Theory, Clarendon, Oxford, UK, 1990.
[7] Arunan, E. *et al*. Definition of the hydrogen bond (IUPAC Recommendations 2011). *Pure Appl Chem* **83**, 1637–1641 (2011).
[8] Popelier, P. L. A. Characterization of a dihydrogen bond on the basis of the electron density. *J Phys Chem A*. **102**, 1873-1878 (1998)
[9]. Popelier, P. L. A. Atoms in molecules. An introduction. Prentice Hall, Harlow, 2000.
[10] AIMAll (Version 15.09.27), Todd, A.; Keith, T. K. Gristmill Software, Overland Park K. S., USA, 2015 (aim.tkgristmill.com)
[11] Koch, U., & Popelier, P. L. A. Characterization of C-H-O hydrogen bonds on the basis of the charge density. *J Phys Chem* **99**, 9747-9754 (1995).
[12] Matthews, R. P. Welton, T & Hunt, P. A. *Phys Chem Chem Phys* **17,** 14437-14453 (2015)
[13] Hunt, P. A. Ashworth, C. R. & Matthews, R. P. Hydrogen bonding in ionic liquids. *Chem Soc Rev* **44**, 1257-1288 (2015).
[14] Johnson, E. R., Keinan, S., Mori-Sánchez, P., Contreras-García, J., Cohen, A. J., Yang, W. Revealing noncovalent interactions. *J. Am. Chem. Soc*. **132**, 6498–6506 (2010).
[15] Contreras-García, *et al*. NCIPLOT: A program for plotting noncovalent interaction regions. *J. Chem. Theory Comput* **7**, 625-632 (2011).
